# Advanced Mobility Handover for Mobile IPv6 Based Wireless Networks

**DOI:** 10.1155/2014/602808

**Published:** 2014-12-31

**Authors:** Ali Safa Sadiq, Norsheila Binti Fisal, Kayhan Zrar Ghafoor, Jaime Lloret

**Affiliations:** ^1^Faculty of Computer Systems and Software Engineering, Universiti Malaysia Pahang, Lebuhraya Tun Razak, Gambang, 26300 Kuantan, Pahang, Malaysia; ^2^UTM MIMOS CoE in Telecommunication Technology Faculty of Electrical Engineering, Universiti Teknologi Malaysia (UTM), 81310 Johor Bahru, Johor, Malaysia; ^3^Faculty of Engineering, University of Koya, Daniel Miterrand Boulevard, Koya KOY45, Kurdistan, Iraq; ^4^Instituto de Investigacion para la Gestion Integrada de Zonas Costeras, Universidad Politecnica de Valencia, 46022 Valencia, Spain

## Abstract

We propose an Advanced Mobility Handover scheme (AMH) in this paper for seamless mobility in MIPv6-based wireless networks. In the proposed scheme, the mobile node utilizes a unique home IPv6 address developed to maintain communication with other corresponding nodes without a care-of-address during the roaming process. The IPv6 address for each MN during the first round of AMH process is uniquely identified by HA using the developed MN-ID field as a global permanent, which is identifying uniquely the IPv6 address of MN. Moreover, a temporary MN-ID is generated by access point each time an MN is associated with a particular AP and temporarily saved in a developed table inside the AP. When employing the AMH scheme, the handover process in the network layer is performed prior to its default time. That is, the mobility handover process in the network layer is tackled by a trigger developed AMH message to the next access point. Thus, a mobile node keeps communicating with the current access point while the network layer handover is executed by the next access point. The mathematical analyses and simulation results show that the proposed scheme performs better as compared with the existing approaches.

## 1. Introduction

Wireless networks are loomed as a promising technology that can provide seamless access to network services. The past decade has witnessed a rapid increase of network services in the Internet at any time and any place. In mobile IP networks, this sharp increase of network services requires seamless mobility and low delay during mobile node's (MN) movement. This can be achieved by a seamless mobility handover management scheme with low signalling cost. Moreover, the quality-of-service (QoS) of ongoing network applications is glaringly affected by mobility handover processes, due to high delay and packet loss. Besides, the support of an efficient mobility management is considered as a one of the very important issues for the future generation of wireless and mobile networks services [1–3].

Basically, the handover signalling process which is used in obtaining the new IPv6 address from visited network (network layer handover) must wait until the link layer handover has performed its own processes. Accordingly, the initiation time is increased and afterward, the overall handover latency is increased similarly. This can be considered as a main reason of degrading the QoS in wireless local area networks (WLANs) [4, 5]. Therefore, the efficient handover process is challenging as a way to achieve a seamless mobility with low handover delay [5–8].

In order to keep the routability between MN and access routers (AR), the IETF has designed Mobile Internet Protocol version 6 (MIPv6) to provide mobility in mobile devices. Within MIPv6 The MN expected to be supplied with IPv6 address whether it is currently connected to the AR belong to its home agent (HA) or other networks with different ARs and configured with different global routing prefixes. Therefore, the MN must obtain a temporary new IPv6 address instead of its* home address* (an IPv6 address assigned by HA of MN which is started with the home subnet prefix) of HA. During the time MN is connected to its HA, all the traffic of packets are addressed to its configured home address. Afterwards, all the packets that were sent by CNs are routed to the MN's HA employing the typical Internet routing mechanisms [9].

On the other hand, when an MN is connected to some foreign networks, it utilizes one or more of care-of-addresses (CoA), that is, depending on the number of visited foreign networks. The assigned CoA is an IPv6 address with a subnet prefix indicating the visited foreign network. By the time MN is active with a particular foreign network, the packets are addressed with the obtained CoA to be reachable to the MN. Moreover, in some cases, MN can accept packets from a number of CoAs, for instance, when it is roaming but still accessible at the previous network.

In MIPv6, there are some issues that still need to be addressed regarding mobility handover process. When MN performs a fast movement, the signal quality of the current access point (AP) can go down rapidly. Thus, MN is not able to connect for a long period with the current AR (CAR), for instance, when MN does not have sufficient time to send handover signals which are responsible for new IP address registration process. Thus, it can lead to mobility handover failure. In other words, the mobility handover process, which is performed by MN to obtain a new CoA from visited network, in addition to the registration processes with HA is negatively affected by inefficient mobility handover process. Consequently it can degrade the QoS of ongoing applications due to high overall handover delay.

In the light of discussions given above, the handover delay is one of the hot issues that are normally experienced within MIPv6 and other well known mobility management protocols [5]. Therefore, it is important to take into consideration this aspect as a way to improve the network layer handover processes. Moreover, the handover delay of several seconds is unacceptable for most of time-sensitive and lost-sensitive applications (real-time applications) [10]. Thus, many mobility handover schemes have been proposed. In this regards, several approaches have been proposed as a way to reduce the handover latency that is experienced within MIPv6 due to IPv6 address configuration and registration processes.

The remainder of the paper is as follows.  Section 2 provides the related literature in mobility handover process of network layer and mobility management approaches, and Section 3 presents the detailed design of proposed AMH scheme, whereas Section 4 illustrates the performance evaluation of proposed AMH scheme followed by the conclusion in Section 5.

## 2. Related Works

One of the essential issues in wireless communications is the handover delay. Hence, many studies have tried to overcome with appropriate solution to decrease the associated time delay during handover process. In order to maintain the mobility handover process of network layer, firstly the Internet Engineering Task Force (IETF) has proposed various IP-based mobility management schemes, such as MIPv6, Hierarchical Mobile IPv6 (HMIPv6), and Fast MIPv6 (FMIPv6) [11–14]. The goal of these schemes is to maintain the communication continuity when an MN moves between different ARs [15, 16]. However, these mobility protocols still have shortcomings in terms of handover latency, packet loss, and signaling overheads [16].

A hierarchical mobile IPv6 (HMIPv6) is proposed by Castelluccia [17] as a way to mitigate the signaling overhead that is caused by the binding update processes. This was achieved by offering some kind of local HA, called mobility anchor point (MAP), in addition to complaining subnetworks into clusters called MAP domains, where each cluster is controlled by a single MAP. However, there are still some issues visible in HMIPv6, for instance, when any single of MAP is failed leads to loss all the communication. Moreover, the performance of real-time applications is not considered in the design phase of HMIPv6 [18].

Furthermore, within HMIPv6, there is a lack of inclusive analysis of different parameters in terms of signaling load. In addition, there is an ignorance of binding refresh cost that is performed after the binding lifetime is expired. On the other hand, the impact of packet tunnelling cost, which could be high due to the high mobility handover delay, was experienced by MN. Thus, many packets could be waiting to be forwarded to the MN after the routability is returned.

As mentioned before, within MIPv6 and HMIPv6 processes there are still some problems such as handover latency, packet loss, signaling overheads, and extensive MIPv6 functionality in the IPv6 load in MN side [19]. Therefore, FMIPv6 [20] has been proposed by IETF as an extension to support the mobility management and improve the performance of MIPv6. This new extension relies on some of the link layer triggers in order to execute network layer handover processes in a fast way [21]. This process is performed by preparing the new IP configuration before the current link breaks down. This was achieved by enhancing the employed ARs as a way to perform a fast binding update (BU) message between previous access router (PAR) and new access router (NAR). Throughout this process the MN was able to send and receive packets once it is attached to the new network link. FMIPv6 assumes that the new CoA (NCoA) configuration messages are received through the current network (the PAR) before the link layer handover is began. This was handled by sending some of network layer signals (which related to the new IP address registration process) during the link layer handover processes to the NAR.

However, FMIPv6 protocol mainly relies on two modes that are reactive and proactive modes, whereby at best the employed anticipation method by proactive mode does not guarantee reliable packet transmission [18]. Moreover, the unpredictable and fast movements that are performed by MN can cause packet loss.

In contrast, a network based mobility management protocol proxy MIPv6 (PMIPv6) [22] was developed as a way to reduce the signaling overhead of MIPv6. Two new elements were introduced by PMIPv6, mobility access gateway (MAG) and local mobility anchor (LMA). The updating process of MN's movement is identified by the MAG, which is a function located at the AR. Thus, MAG updates to the LMA which is to perform the functions of HA in addition to managing the routing process of data packet to the MN. Subsequently, the MN was able to change its locations without any signaling generated by MN's side. When the MN is registered at the LMA, the same home network prefix (HNP) is provided by LMA to the MN. The HNP is sent by employing a proxy binding acknowledgement message, which responds to a proxy binding update message sent from the MAG.

However, PMIPv6 was developed mainly for the local mobility management, which does not support a global mobility management [15, 23, 24]. For instance, when some MNs support only MIPv6 and intend to use PMIPv6, the network infrastructure should provide MIPv6 functions to them. Therefore, the MAG in this case cannot act like the HA. The MAG is supposed to advertise its own routing prefix instead of the IPv6 prefix that the MN always receives when connected to any AP within PMIPv6 domain (MN-HoA). Accordingly, these MNs without implementation of PMIPv6 need to rely on the network to manage their mobility. Hence, it can be noticed that the PMIPv6 is facing scalability issues when MNs from global network prefixes enter its network domains.

Although, some approaches recently are tried to enhance the functionality of PMIPv6, until now they could not efficiently support the seamless mobility handover [15, 16]. The reason is that within PMIPv6 a high handover delay is normally experienced by intersubnet handover. Thus, high packet loss could occurr especially with real-time applications due to the high packet rate. Moreover, in case of any single failure of a MAG can lead to degrade the performance to be worst than standard MIPv6.

In order to cope with the issues that normally occurred by adding the authentication, authorization, and accounting (AAA) functions with the MIPv6 handover processes, such as the high cost delay of authentication and registration process, the authors in [25] developed a novel hierarchical authentication scheme. The proposed method focused on minimizing the authentication latency in AAA processing. In the proposed scheme, a hierarchical AAA architecture was achieved, in which the AAA servers are deployed on the MAP. The authors analyzed the signaling cost of the mobility handover process of the proposed authentication registration (AuthR) method.

AuthR method is considered the total cost of mobility handover process, *C*_total_, which contains three parts, the transmission cost of registration signaling (*C*_reg_), the transmission cost of authentication signaling (*C*_auth_), and packet delivery cost (*C*_trans⁡_). The costs of registration and authentication signaling are considered together in the proposed AuthR method, which is called *C*_RA_. Furthermore, *C*_trans⁡_ was indicating the packet processing and delivery cost during mobility handover process. Total cost of mobility handover process was given by

(1) $$\begin{array}{c} C_{\text{total}}=C_{\text{reg}}+C_{\text{auth}}+C_{\mathrm{trans}}=C_{\text{RA}}+C_{\mathrm{trans}}. \end{array}$$

However, it can be observed from Formula (1) that the authors did not consider the signalling delay and overhead of registration and address configuration processes. In other words, the registration and address configuration signaling overheads can contribute in increasing the total handover delay. On the other hand, due to security issues that could be araised when MNs perform many changes of their positions throughout the IPv6 network while retaining their existing connections these processes need to support low delay time [26], many advantages are thus attained, but obtaining low signalling overhead or cost with considering security aspects remains a fundamental concern [27].

An improved fast handover scheme for hierarchical mobile IPv6 (IF-HMIPv6) was proposed in [28], in order to reduce the handover delay and packet loss. By the time the MN sent an improved router solicitation for proxy (IRtSolPr) message to the MAP, the procedures of IF-HMIP method began during the link layer handover process. The IRtSolPr message contains information about the MAC address or identifier of the AP. Moreover, as a way to identify the MAP that MN follows during IF-HMIPv6 processes, an “I flag” in IRtSolPr message was included. By using this “I flag,” MAP was able to process the new CoA configuration process on behalf of the MN and sent the handover initiation (HI) message to the NAR in order to establish a tunnel.

Consequently, the MAP sent the proxy router advertisement (PrRtAdv) message to the MN as a response to the “I flag,” which was sent via IRtSolPr message of MN as an indicator for handover initiation processes. Thus, MN is waiting for the FBAck instead of sending the F-BU to the MAP before it received the F-BAck. The duplicated address detection (DAD) process was processed by NAR to verify the new CoA. DAD process was performed after NAR is received and the HI sent by MN. When DAD process is verified by the new CoA, a response with a handover acknowledgement message (HAck) was sent by AR to MN. Eventually, the tunnel is established between MAP and NAR.

Furthermore, an evaluation based on numerical model of proposed IF-HMIPv6 was conducted during the study. Formula (2) shows that the time delay within one MAP is represented by L2's delay only, whereas Formula (3) illustrates the time delay that occurred during handover processes between different MAPs, where *T*_BU(*M*-HA)_ is the time, is required to perform binding update to HA by local MAP. Consider the following:

(2) $$\begin{array}{c} T_{\text{IF-HMIP-inter}}=T_{L2}, \end{array}$$

(3) $$\begin{array}{c} T_{\text{IF-HMIP-inter}}=T_{L2}+T_{\text{detect}}+T_{\text{CoA}}+T_{\text{BU}(M\text{-HA})}. \end{array}$$

However, it can be observed from Formula (3) that the time was consumed in address configuration process of CoA inside one local MAP that is still visible. Moreover, this study did not discuss the consequences behind increasing the number of MNs. In other words, the negative effects which lead to signaling overhead then decrease the QoS on going applications due to high number of MNs.

On the other hand, a seamless flow mobility management architecture (SFMMA) for vehicular communication networks was proposed in [29]. The proposed SFMMA method was introduced as a way to cope with the mobility management aspects in vehicular ad hoc networks (VANETs), in order to maintain the wireless connection with roadside network service nodes during vehicular movement. The authors in [29] are designed a multiaccess wireless network architecture employing the Wi-Fi (WLANs), vehicular, worldwide interoperability for microwave access (WiMAX), and long-term evolution 4G (LTE) technologies, supporting a continuous and wireless connection for the ongoing vehicular applications. This method was devised for the needs of each application class, such as throughput, delay, and packet loss. The status of the current active wireless connection was considered in order to tackle the division of the mobility flow. Furthermore, the authors elaborated the handover decision using three elements that are based on proxy MIPv6 (PMIPv6), the local mobility anchor (LMA), the mobility access gateway (MAG), and MN.

However, at best, by using the SFMMA scheme MN still asking information from the MAG where the network interface is connected; inquires about network status and the home network prefixes (HNP). Throughout these amounts of information, MN can indicate whether the interface is still active or not; otherwise it does not know to whom it needs to route the packets and what are the prefixes that can be used to send its packets. Furthermore, the triggering process of mobility handover decision can be performed by many sides as it was stated in the proposed network architecture, which are MN, LMA, and MAG. These kinds of decisions can lead MN to experience unnecessary handovers [30]. The reason is that when a rapid changing occurrs with MN's position due to roaming process, many mobility aspects are changeable that in such circumstances (high speed) it takes time in updating the parameters with both LMA and MAGs. Therefore, it could be more difficult to include all MNs, LMAs, and MAGs in mobility handover decision making processes that lead to unstable handover decision. Besides, this can result in an increase in the signaling costs and overheads of the entire handover processes.

A host-based localized mobility management (LMM) scheme was proposed by Cho et al. [5]. The developed scheme supported multiple care of addresses and fast handover mechanism. The handover processes within LMM scheme are controlled using a developed localized mobility client (LMC) server. The connection was established by creating a tunnel by LMC as a way to process the location registration. A BU message was sent using tunnel in addition to handle the MN communication over this tunnel. After the link is established with candidate network, the CoA is formed to be assigned for MN.

However, the proposed scheme still offers complexity in network handover signaling process. This due to the fact that introducing localized mobility management clients, servers, and Tunnel Gateways (TG); with any failure of one of them it degrades the whole system performance. Moreover, it can be noted that the MN still engages with many of control signals as it was demonstrated in [5]. In other words, the tunnel establishment trigger and the BU messages are still visible within MN's side, which keeps the overheads over MN during mobility handover processes. Furthermore, MNs still inquire CoA from visited networks in order to obtain the IP connectivity, which also requires more time delay.

## 3. Advanced Mobility Handover Scheme (AMH)

**Figure 1:**
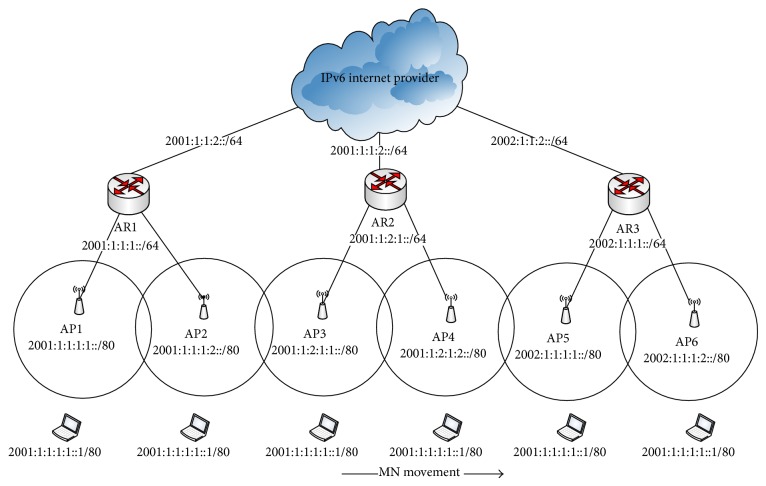
Proposed MIPv6 addressing scenario for AMH.

In this section the detailed design of the AMH scheme is discussed by illustrating perspective architecture of AMH to explain how the handover process is performed. After the handover decision with one particular AP is taken, the AMH scheme will be triggered by MN. The triggering message is enclosed in an association request frame during the IEEE 802.11/b handover process. The AMH process is triggered as a way to inform the HA and CN the changes in the network layer (the changes with associated AR) of the wireless networks through the new selected AP. In AMH architecture there are ARs connected to the Internet service provider (ISP) which relies on IPv6, APs, and MN being mobile across the deployed APs which is illustrated in Figure 1. These APs can access the Internet connection through ARs. Figure 1 shows the mobility scenario when MN crosses different APs and different ARs. The internal configured subnet addresses for AR1, AR2, and AR3 are (2001:1:1:1:/64), (2001:1:2:1:/64), and (2002:1:1:1:/64), respectively. These addresses are identifying three different subnets, whereby the global routing link is configured with the same routing prefixes for both AR1 and AR2, whereas AR3 with different routing prefix of (2002:1:1:2:/64). Thus, the main difference between AHM2 and AMH3 is that, when an MN moving from AP2 to AP3, the subnet IPv6 address within connected AR is changed as well as the APs IPv6 address, but the global routing prefix of global connection is still the same for both AR1 and AR2; this is during AMH2 process. On the other hand, in AMH3 both IPv6 addresses of subnet of AR and global routing connection are different. Hence the presented processes by AMH3 design will be implemented to tackle this scenario.

During the roaming process the MN uses a unique home IPv6 address assigned by HA in order to keep traffic flowing without a CoA. Therefore, the proposed AMH scheme introduces a unique IPv6 address which can be used by the MN during the roaming process. The introduced unique IPv6 address was based on standard 128-bit IPv6, which can be used by the MN during the roaming process for global mobility purposes. It is worth mentioning that the proposed unique local IPv6 unicast addresses by [31] could not achieve the globality in maintaining the network connection using the obtained unique IPv6 addresses [32].

These unique IPv6 addresses in AMH scheme were achieved by allocating three fields which are global routing prefix field (with high probability of uniqueness), AP-ID, and MN-ID fields in the IPv6 address of any MN, AR, and AP. The IPv6 addresses have been configured in each AP by first assigning the global routing prefix, which is a field of the IPv6 address indicating the APs that are connected to the same AR. The second developed field of the AP IPv6 address is the AP-ID which uniquely identifies each AP and the third developed field of the IPv6 address is the MN-ID in AP, which is assigned to zero in the IPv6 addresses of APs and ARs. On the other hand, the IPv6 address for each MN during the first round of AMH process is uniquely identified by HA using the MN-ID field. Moreover, another temporary MN-ID_temp_ is generated by AP in each time an MN was associated with that particular AP and temporarily saved in a developed table inside the AP.

The first step of configuring the proposed unique IPv6 is creating the global routing prefix field. The bit length that utilized to configure this field was assumed to be 64 bits, because the availability of high utilization ratio does not require internal structure based on [31]. Therefore, this allocation was used to achieve high probability of uniqueness and avoid any clash situation with other assigned IPv6 addresses to an MN in the structured wireless network. The pseudorandom method [33] was employed in allocating the ID number of global routing prefix field during each time the unique IPv6 address is generated. Apart from this method, the way of generating the ID number of a particular global routing prefix is not sequential but is randomly identified with high probability of uniqueness. Similarly, the same pseudorandom method was used in obtaining the unique MN-IDs and MN-ID_temp_, which afterwards were utilized in global routing purposes when the MNs visiting an AR configured with IPv6 with different global routing prefix (more explanation about this process was discussed by AMH3 processes in Section 3.3). On the other hand, the AP-ID of the deployed APs in simulated scenario was configured uniquely as a way to avoid the conflicts that might occurred among them. AP-ID field was set to *i* equals to 16 bits in the proposed IPv6 format.

The probability of proposed unique IPv6 address to be clashed with other assigned MN's unique IPv6 addresses was given by

(4) $$\begin{array}{c} P_{\text{unique}\;\;\text{IPv}6\;\;\text{collision}}=1-\exp\left(\frac{-{\text{MN}}_{\text{number}}^{2}}{2^{\left({\text{GRP}}_{L}\right)}}\right), \end{array}$$

where MN_number_ is the number of MNs that assigned with unique IPv6 addresses and GRP_*L*_ is the bit length of global routing prefix field. Thus, for instance, when the number of associated MNs is 10000 and the GRP_*L*_ is 64-bits; the *P*_unique  IPv6  collision_ equals 2.71 × 10^−12^ which infers a very low probability of collision.

**Figure 2:**
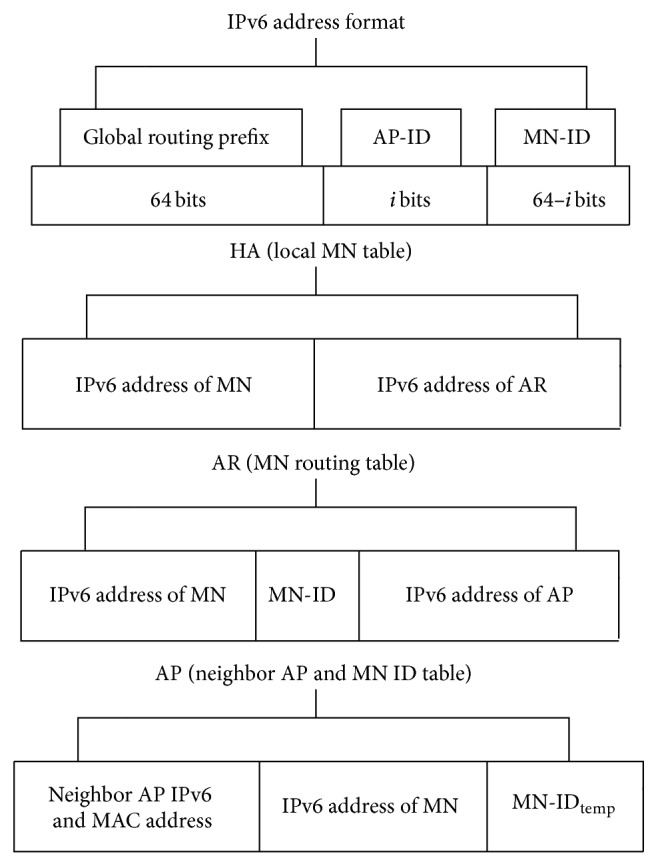
The format of the proposed unique IPv6 address and data structure as a table form saving in each of HA, AR, and AP in the proposed AMH scheme.

Figure 2 shows the IPv6 address format and data structure in table form saved in each HA, AR, and AP in the proposed AMH scheme. Three IPv6 fields (IPv6 address format) are illustrated in Figure 2 where *i* is set to 16 bits. Moreover, the data structure in table form is shown in Figure 2. This table is designed and integrated into the proposed AMH in order to ensure that the IPv6 address of AP and AR during the MN roaming process is updated. These tables are utilized in AP, AR, and HA. Figure 2 shows the data structure in table form, known as (local MN table), which has been saved in HA. This local MN table in HA contains the MN's IPv6 address along with the IPv6 address of the AR that the MN is currently connected as shown in Figure 2. The routing table in each AR (MN routing table) consists of the IPv6 address of the MN with MN's ID in case the MN is attached to an AR with a different global routing prefix and AP's IPv6 address where the MN is currently associated with as shown in Figure 2. Finally, in each particular AP the table of the neighbor of AP's IPv6 and MAC address, current associated MNs IPv6 addresses, and MN's ID for each MN created by AP after the association process are saved as a table as shown in Figure 2.

**Figure 3:**
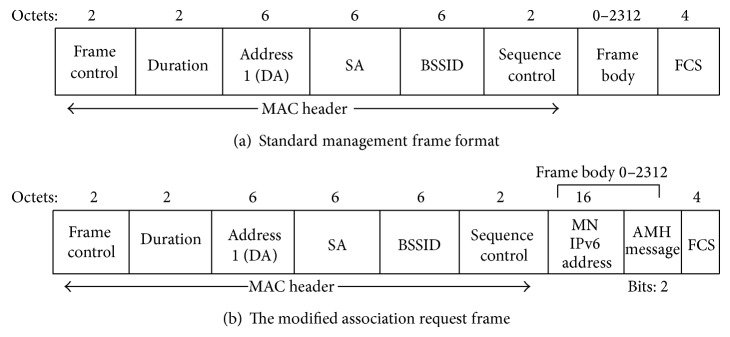
The management frame format and modified association request frame.

In order for the MN to trigger the AMH scheme in the time the handover decision has been taken, the AMH message has been developed. In standard 802.11/b there are various frame types defined to be used by MN and AP for communications, as well as managing and controlling the wireless link. The association request frame in Standard 802.11/b is sent by MN to AP in order to allocate resources for the MN and to synchronize with a radio network interface card (NIC). The association request frame is a management frame as defined in [34]. As illustrated in Figure 3(a), DA is the destination address, SA source address, and BSSID basic service set ID which is equivalent to AP ID and FCS frame check sequence. Basically, the frame body of a management frame of subtype association request contains information about the MN such as supported data rates, the SSID of the network the MN intends to associate with, and some other information. In the proposed AMH scheme the association request frame's body is modified by adding 130 bits. The IPv6 address of the MN uses 128 bits in each association frame plus 2 bits for the AMH triggering the AMH message.

In the proposed AMH architecture three handover cases are considered which are handovers within the same subnet (AMH1), with different subnets and same global routing prefix (AMH2) and with different subnets and different global routing prefix (AMH3).  Figure 3(b) depicts the modifications that have been done on the association request frame that the MN sends to AP. The IPv6 address of the MN is included in the body of association request frame each time a request is sent to the new selected AP. Moreover, the AMH message code included in each association request frame is sent by the MN in order to trigger the AMH scheme by the selected AP.  Table 1 shows the three types of association request frame sent by the MN and the AMH procedure which is executed by the next AP. When the MN includes the AMH code (01) a request is made of the selected AP to reserve memory space and establish the MN-ID_temp_ which will later be used as a route distinguisher of the packets which are directed to the MN. Thus, this created MN-ID_temp_ will be used to send and receive traffic between the MN and AP.

Moreover, when the AP receives the AMH code (01) the AMH1 procedure will be triggered in order to perform the mobility handover on behalf of the MN. In addition, when AP receives an association request frame from the MN, it includes AMH code (10), which means repeating the previous procedure with AMH2 process. At the same time if the received AMH code sent by MN is (11), AP will handle the AMH3 procedure. This AMH message code is placed in the two modified bits in the association request frame as illustrated in Figure 3(b). These three different AMH handover procedures AMH1, AMH2, AMH3 are discussed in detail by the following sections with practical examples of table entries updated in AP, AR, HA, and CN.

### 3.1. Advanced Mobility Handover Scheme within the Same Subnet (AMH1)

**Figure 4:**
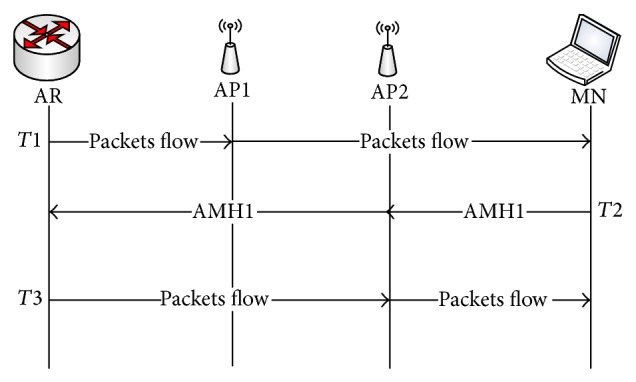
AMH1 scheme within the same subnet.

Two APs are considered to be in the same subnet when both of them are connected to the same AR (that belong to one subnet). In this scenario, when the MN moves from current AP to the next, the RSS is gradually decreasing with the current AP in which communication is to continue. At this point in time, which is called passive scanning, the MN receives beacon frames from the next AP in the vicinity. Basically, beacon frames are periodically transmitted by the AP to announce the presence of a WLAN network. Through these beacon frames, MN can extract the next AP's IPv6 address using the obtained new AP MAC address by searching for it in the created neighbor AP's IPv6 list of current APs, which is presented by the table structure of AP in Figure 2. Utilizing this process, the MN realizes whether the next AP belongs to the same current subnet or not. This process is performed during time point *T*1 as shown in Figure 4. Thus, using this obtained IP address the MN can determine if any changes have occurred with the subnet using AP-ID which is uniquely identified by each AR for associated APs as mentioned before. In case the next AP is attached to the same subnet, the MN sends the AMH1 message code included in the association request frame to the next AP (AP2) as shown in Figure 4, asking that the AMH1 handover process be triggered during period *T*2.

Figure 4, illustrates the execution procedure of AMH1 scheme within the same subnet. As depicted in the figure, the MN sends the AMH1 a message which contains its IPv6 address along with code for AMH1 using the association request frame to the AP2. When the next AP (AP2) receives this frame it performs the default process of association request in addition to establishing the new MN-ID_temp_ that will be used by AP in packet forwarding processes. The MN-ID_temp_ establishment process was done by AP, utilizing the reserved 16 bits within the developed unique IPv6 address which is allocated on MN-ID's field. When an AP accepted MN's association request, it creates an ID which is represented by a decimal number to uniquely identify the MN, which is placed on the 16 bits of MN-ID field. AP then forwards AMH1 message to the AR. This AMH1 message sent by AP2 contains the IPv6 address of the MN and IPv6 address of the AP2. After AR receives the AMH1 message coming from AP2, it checks its MN routing table and updates the IPv6 address to be the IPv6 address of AP2 in the routing entry. It can be observed that during the time consumed by the AMH1 scheme, the MN kept the connection to AP1 in the link layer. Therefore, it could receive the data forwarded by AP1. Thus, the MN is still able to receive the data destined and forwarded to the home IPv6 address even though the link attachment changed. Afterward, when AMH1 has been completed, the MN disconnected from the AP1 in the link layer and started to receive data from the AP2 in time *T*3. Thus, the AMH1 ensures packet flow between the MN and AR during the communication as shown in Figure 4.

Table 2 shows the data updating in AP2 and AR1 which are illustrated in Figure 1 as a practical example for AMH1. This presented example shows that when the MN moves from AP1 towards AP2 both are associated to AR1. The IPv6 address of the MN has been added in AP with MN's ID created by AP2 which is (1) in this present example. After AMH1 is received by AR1, the routing table is updated by replacing the IPv6 address of AP1 with the obtained IPv6 address of AP2, to be placed as the new active AP for MN with IPv6 address of (2001:1:1:1:1::1/80). Thus, when the packet traffic was sent to AR1 forward it to the new AP's IPv6 address of AP2.

### 3.2. Advanced Mobility Handover Scheme within Different Subnets and the Same Global Routing Prefix (AMH2)

**Figure 5:**
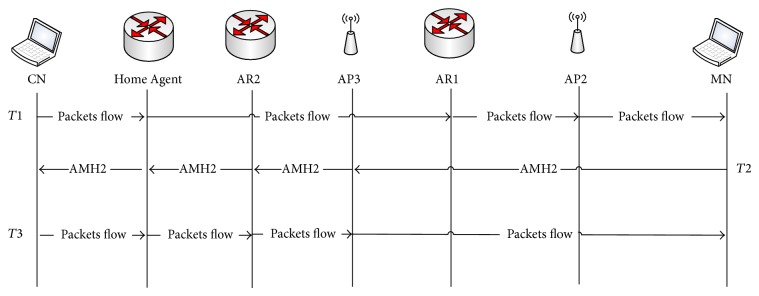
AMH2 scheme within different subnets and the same global routing prefix.

As mentioned earlier the MN can extract all information related to the next AP through its beacon frame during time point *T*1. When the MN realizes that the next AP belongs to a different subnet but the global routing prefix is the same as the current AP, the AMH2 scheme for different subnets and the same global routing prefix will be triggered in *T*2.  Figure 5 shows that the MN sends the AMH2 message to the next AP (AP3) to trigger AMH2 while the MN is still communicating with the current AP (AP2). When AP3 receives the AMH2 message sent by the MN, the AP3 creates the MN-ID_temp_ and then forwards AMH2 to the associated AR2 which contains the MN and AP3 IPv6 address. Once the AMH2 message reaches AR2, the routing table will be updated by adding the IPv6 address of AP3 and that of the MN which it is intending to communicate with. The AMH2 message will then be forwarded via AR2 to the HA of the MN through the Internet and the payload of this message will include the IPv6 address of the MN and the IPv6 address of AR2.

After receiving AMH2 the HA then updates the IPv6 address of the associated AR to the address of AR2 and forwards the AMH2 message to the CN which contains the IPv6 address of the MN and AR2. Moreover, AR2 established the tunnel between itself and AR1 as a way to forward all the cached packets' flow that was visible in *T*3. It is worth mentioning that the tunnel establishment order that was triggered by NAR is based on the code presented within AMH2 message (10). The reason of this tunnel is to ensure reroute and old packets that were destined to the previous AR before mobility handover was completed with NAR. Thus, those buffered packets will be delivered to MN via NAR after it was physically connected to NAR. Eventually, when CN received the AMH2 message it updated its routing entry with the new IPv6 address of AR2 in order to retain the routability with the MN through the newly visited AR2 with a different subnet but the same global routing prefix.  Figure 5 also shows that during the time of AMH2 processes, the MN was still connected to AP2 in the link layer. Therefore, the packet flow forwarded by CN and HA through AP2 can still be received and data continuity has been ensured using AMH2. Figure 1 illustrates the presented AMH2 process scenario when the MN was moved from AP2 that belongs to AR1 toward AP3 that is associated with AR2.

Table 3, shows the practical example of data updating in AP, AR, HA, and CN after AMH2 message was triggered by the MN. It can be observed that the AP's IPv6 address in AR2 is updated with AP3 address (2001:1:2:1:1::/80) in which the MN will use to communicate with during time *T*3. Afterwards, the routing tables of HA and CN are updated by a new AR entry, (IPv6 address of AR3) which it obtained after sending AMH2 message from AR3 to HA and CN through the internet. Thus, all the packets that are forwarded to MN will be redirected to the new visited AR3 during *T*3 using the established tunnel between AR2 and AR1. In other words, the IPv6 address was updated in AR, HA, and CN after the MN triggered an AMH2 message with code (10) to AP3 during time *T*2 while still connected to AP2 that belongs to AR1, as can be seen in Figure 5. Subsequently, the advanced mobility handover was tackled via AP3 on behalf of the MN which allows the MN to save the time normally consumed for this process to be performed prior to its default time (after link layer handover is completed). Hence, MN through the proposed AMH scheme could achieve an efficient IPv6 address configuration and registration process.

### 3.3. Advanced Mobility Handover Scheme within Different Subnets and Different Global Routing Prefixes (AMH3)

AMH3 has been developed in order to use the AMH scheme to tackle the handover when the MN visits a new network with a different global routing prefix. More precisely, when the visited networks are configured with a different global routing prefix the MN sends AMH3 message to the newly selected AP which is associated with that visited network. The AMH3 message is included in the association request frame which contains code (11) along with the MN's IPv6 address as shown in Table 1. As mentioned before in AMH1 and AMH2 the AP creates the MN's ID once the AMH message is received (i.e., AMH3). Afterwards, it forwards AMH3 to the associated AR which includes the MN's IPv6 address, MN's ID, and new AP's IPv6 address. When AR receives the AMH3 message, the default home network IPv6 address that the MN's IPv6 address belongs to is extracted using (AND) process with the unique IPv6 address of MN that was sent via AMH3 message using the global routing prefix of the MN.

Thus, the NAR which had a different global routing prefix used the extracted home network IPv6 address to send AMH3 through Internet to HA of MN. The AMH3 message sent by AR included the MN's IPv6 address and the IPv6 address of NAR with a different global routing prefix. Moreover, the IPv6 address of the new AP along with MN's ID is added as a routing entry of the new AR. In other words, the global routing prefix of visited AR is added to the home subnet interface. The AMH3 message was forwarded to CN by HA in order to inform the new IPv6 address of the newly visited AR to which the MN is currently connected. Moreover, the IPv6 address of the new AP along with MN's ID is added as a routing entry of the new AR. In other words, the global routing prefix of visited AR is added to the home subnet interface. Thus, the routing table entry of CN was updated by searching MN's IPv6 address then replacing the current active AR's IPv6 address. After the handover was initiated with new selected AP and after the mobility handover has been performed with that selected AP, the previous AP is informed that the MN is physically disconnected after the disassociation frame is invoked by MN informing AP to canceling its previous session with it; otherwise it will be automatically disassociated by AP after certain timer is over due to no active session is between AP and MN.

**Figure 6:**
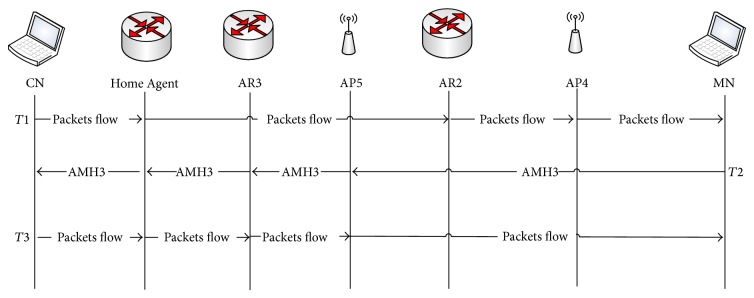
AMH3 scheme within different subnets and different global routing prefixes.

On the other hand, after the mobility handover was done with new AR (NAR) the NAR is initiated neighbor advertisement message informing the previous AR of the existence of MN (MN is currently under its routing table). Thus, the tunnel is established between NAR and previous AR. The process of exchanging AMH3 messages between NAP and ARs continued until the updated AMH3 message reached the CN. These processes occurred during the time point of *T*2 as illustrated in Figure 6.

In order to shed the light more on the packet flow directions, another scenario of communication is presented that we called down-link communications (CN-to-MN). Therefore, in time point *T*3 when packets' flow returned from CN to MN, the packets were encapsulated with the new IPv6 address of the new AR with a different global routing prefix. When AR receives the packet flow which was forwarded to the MN, AR sent it to the new AP's IPv6 address that the MN is currently attached to using the MN's ID that is equipped in an AMH3 message sent by NAP. In order to forward the packet flow by NAP, the MN's ID which was created by NAP is used to send the packets to MN. In other words, the AR and AP used the MN's ID in order to forward the packet flow to the MN instead of the IPv6 address with a different global routing prefix. Thus, the MN within different subnets and global routing prefixes could keep the proposed unique home IPv6 address without requesting a CoA from each new visited network by using the AMH3 scheme.

A practical example based on the illustrated scenario in Figure 1 is presented in this paragraph. The updating of the routing table entry data during an AMH3 process in AP, AR, HA, and CN is shown in Table 4. When the MN moved from AP4 which belongs to AR2 towards AP5 which belongs to AR3 with a different subnet and global routing prefix (2002:1:1:1), the MN sent AMH3 including its IPv6 address with an AMH3 code (11). The AP has included the MN's IPv6 address along with an established MN's ID and its IPv6 address is to be updated as an entry in the AR routing table. By the time the AMH3 message reached HA, the IPv6 address of the MN has searched its routing table and the current associated AR has been updated from AR2 to AR3 IPv6 address (2002:1:1:1::/64) as shown in Table 4. The same process occurred in CN when the AMH3 message from HA was received.

## 4. Performance Evaluation

### 4.1. The Numerical Model for AMH

In order to prove the efficiency of the AMH scheme, a numerical model has been created to show the delay time and the cost during handover procedure. In general the mobility handover delay time, *T*_delay_, of MN includes the handover detection, handover decision, and handover execution delay time. The *T*_delay_ can be extracted as in the following form:

(5) $$\begin{array}{c} T_{\text{delay}}=T_{\text{detection}}+T_{\text{decision}}+T_{\text{execution}}, \end{array}$$

where *T*_detection_ in AMH approach can be calculated as follows:

(6) $$\begin{array}{c} T_{\text{detection}}=T_{\text{Addr-Config}}+T_{\text{Home-Reg}}. \end{array}$$

Formula (6) shows the time taken by MN in order to detect the next AP. The time of *T*_Addr-Config_ and *T*_Home-Reg_ is defined as the delay time taken during the BU message exchange between the previous AP and the HA. Since in the AMH scheme the *T*_Addr-Config_ and *T*_Home-Reg_ have been removed using the unique IPv6 address assigned by HA, the AMH messaging process is to be performed between the next AP and next AR and then forwarded through the Internet to the HA. Therefore, the MN informs HA and CN to upload the attached AR's IPv6 using AMH message while still within the current AP coverage area. Formula (7) shows the handover delay time obtained by utilizing the AMH scheme. Consider

(7) $$\begin{array}{c} \text{Handover}-T_{\text{delay}}=T_{\text{decision}}+T_{\text{execution}}. \end{array}$$

To analyze the signaling cost in the proposed AMH scheme, the numerical model described below is used.

The total cost in MIPv6 within the same subnet is calculated in Formula (8). Since the IPv6 address of MN remains unchanged within the same subnet during the handover process, the *C*_Add-Config_ is eliminated from the signaling cost in inner domain scenario:

(8) $$\begin{array}{c} C_{\text{Total}}=C_{\text{Detection}}+C_{\text{Add-Config}}+C_{\text{Reg}}. \end{array}$$

On the other hand, Formula (9) calculates the signaling cost in AMH scheme in inner domain. For the duration of the handover within the same subnet, signaling cost includes the mobility detection cost *C*_Detection_ and the registration cost *C*_Reg_Inner__ which is handled by next AP in AMH scheme using AMH message sent by MN to trigger the handover. Hence, the *C*_Reg_Inner__ has been eliminated from the MN side in the proposed AMH scheme as shown in Formula (10). Consider

(9) $$\begin{array}{c} C_{\text{Inner}}=C_{\text{Detection}}+C_{{\text{Reg}}_{\text{Inner}}}, \end{array}$$

(10) $$\begin{array}{c} C_{\text{Inner}}=C_{\text{Detection}}. \end{array}$$

In contrast, the signaling cost between different subnets and global routing prefix *C*_Outer_ contains the mobility detection cost *C*_Detection_, the registration cost *C*_Reg_Outer__ between subnets, and the cost of the tunnel between NARs and CARs *C*_Tunnel_ as was illustrated in formula (11). In the proposed AMH scheme, the MN was sending AMH2 and AMH3 employing association request frame to the next AP, and the *C*_Reg_Outer__ has been dealt with by NAP instead of MN. Therefore, the *C*_Reg_Outer__ was omitted from the MN side in the outer domain as well. Formula (12) shows the calculations of the *C*_Outer_ in AMH scheme. Consider

(11) $$\begin{array}{c} C_{\text{Outer}}=C_{\text{Detection}}+C_{{\text{Reg}}_{\text{Outer}}}+C_{\text{Tannel}}, \end{array}$$

(12) $$\begin{array}{c} C_{\text{Outer}}=C_{\text{Detection}}+C_{\text{Tannel}}. \end{array}$$

It can be determined from Formulas (10) and (12) that the address configuration cost *C*_Add-Config_ which is defined as the cost consumed by the MN to obtain the CoA from NAR and *C*_Reg_ to register the change of MN and AR IPv6 address with HA does not exist. This is due to the fact that the MN is configured with a unique IPv6 address in the proposed AMH scheme without the need to obtain a CoA from visited networks. Moreover, during the handover process the MN informs the newly selected AP through the association request frame to handle the registration process with NAR then forward to HA using AMH messages. Thus, the *C*_Add-Config_ and *C*_Reg_ in the AMH scheme are eliminated from the MN side which contributes to decreasing the total handover cost.

### 4.2. Simulation Setup

In this section, we present the simulation setup to validate and evaluate the proposed AMH scheme. The AMH scheme was modelled and simulated using an INET framework, which is an open-source communication networks simulation package for the OMNeT++ simulation environment. An extensible Mobile IPv6 (xMIPV6) [35] was utilized in order to design and model the proposed AMH scheme that was based on MIPv6. The network layer architectures of xMIPv6 within each of the MN, AP, AR, and HA was modified as a way to maintain the functionality of the proposed AMH scheme during the mobility handover processes.

The TCP/IP used five layers for the design of the network communication architecture for the AMH scheme. The area of the simulation scenario was 2300 × 1300 meters and was configured with OMNeT++ simulation based on a map of the Campus of the University of Erlangen-Nuremberg. In addition, the simulation scenario was executed using an Intel(R) Dual-Core CPU 2.40 GHz with OMNeT++ 4.3 simulator software. In the simulated scenario, five different subnets were created that were all based on MIPv6 protocol in the network layer, to evaluate the proposed AMH. Two of these subnets were configured with different global routing prefixes to evaluate the AMH3 subscheme.

In general, each particular subnet was represented by one AR classified as AR1, AR2, or AR3. AR4 and AR5 represented different global routing prefixes used to compare with the other routing prefixes. Two APs were connected to each AR, except for AR5, which was connected with only one AP. Furthermore, each AP function had a transmitted power of 14 dbm and radio coverage that covered 300 meters. The distance between two APs was 500 meters resulting in approximately 20% of overlapped area. It is worth mentioning that the Internet connections for the ARs were all based on IPv6 addressing.

**Figure 7:**
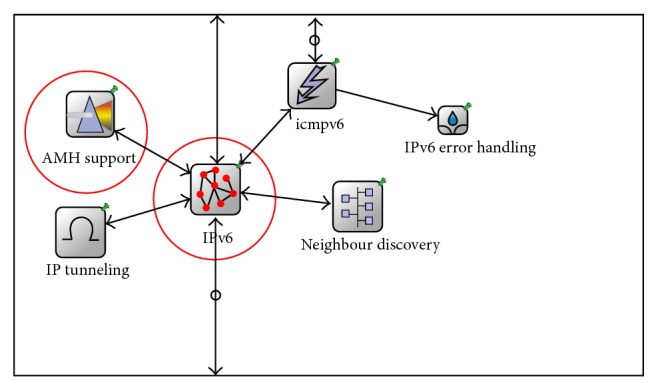
Architecture of the AMH scheme network layer.

Figure 7 shows the architectural design of the network layer of the proposed AMH scheme that was designed and configured using OMNeT++. The modified modules are highlighted with red circles. All the AMH supported algorithms were implemented in the AMHsupport module, which was the modified ipv6 module.

**Figure 8:**
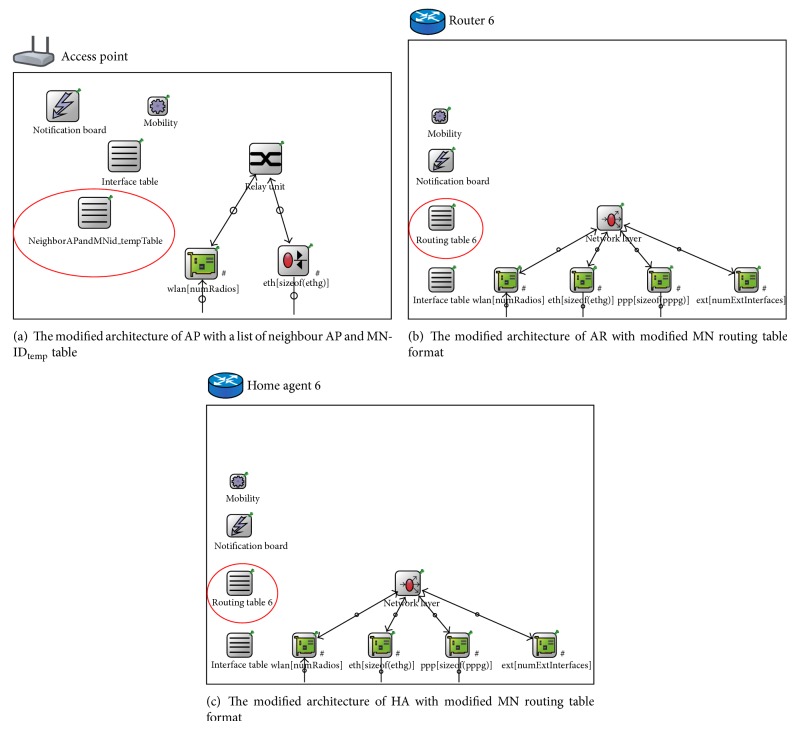
Architecture design of link and network layers of AP, AR, and HA with modified AMH tables.

Figure 8 shows the underlying design of the link and network layers of the AP, AR, and HA in the simulated scenario.  Figure 8(a) presents the architectural design of an AP in the simulated scenario. The highlighted neighbour AP and MN-ID_temp_ table was created in that AP. Figures 8(b) and 8(c) highlight the modified routing tables within AR and HA architecture.

The simulated scenario contains 50 MNs, during the first round of simulation they are distributed as follows: Subnet 1, Subnet 2, Subnet 3, and Subnet 4 with 11 MNs in each, while Subnets 5 with 6 MNs. Each MN moves with speed of 1–18 meter/second and starts from the subnet that belongs to which considered as its HA across the other different ARs. The linear and rectangle mobility models were used to generate mobility in the simulated scenario for the AMH scheme. Update intervals of mobility speed that ranged from 0.1 to 1 seconds were configured by the implemented mobility models for each MN. During the roaming process MN communicates with CNs. The traffic has been generated between MNs in simulation scenario and CN as four types of applications HTTP, FTP, VoIP, and video. The traffic model for each application runs between MNs and CN are listed in Tables 5, 6, 7 and 8.

The MAC layer of the AMH simulation scenario was configured based on IEEE Standard 802.11b, where the PCF was used to simulate the MAC layer of the deployed APs. The channel bandwidth that was used in the simulation was configured to 11 Mbps [36]. In order to set the neighbour discovery process, an interval in the range between the minimum router advertisement interval (minIntervalBetweenRAs = 0.03 s) and the maximum router advertisement interval (maxIntervalBetweenRAs = 0.07 s) [9] was configured. While the wireless channel settings of WLAN were configured, the passive and active scan processes were activated with a probe delay of 0.1 second and minimum and maximum scanning times of minChannelTime = 0.15 second and maxChannelTime = 0.3 second, respectively. The authentication and association time-outs were configured to 5 seconds. The maximum size of the higher layer data buffer in bits was specified as 256000 bits.

**Figure 9:**
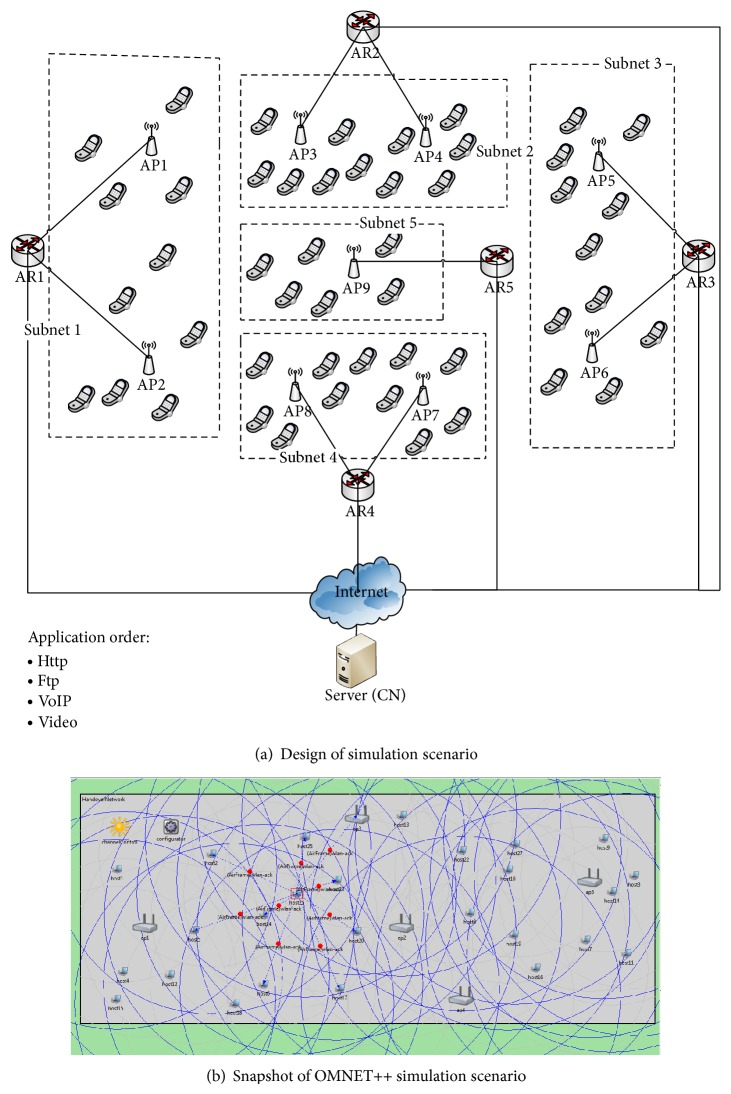
Simulation scenario.

The maximum packet generation rate set to 800 packets/second with maximum packet size of 1000 bytes in the simulated scenario. The simulation time was set to 700 seconds and the applications start time after 100 seconds of simulation time as a way to avoid the effect of transient behaviour on the simulation results. The simulation parameters are listed as shown in Table 9.  Figure 9(b) shows the snapshot of the simulated scenario in OMNeT++.

The received power fluctuates with log-normal distribution about the mean distance-dependent value [37]. The shadowing model is given by

(13) $$\begin{array}{c} \text{PL}\left(d\right)\left[dB\right]=\text{PL}\left(d_{0}\right)+10\times \upsilon \times \log\frac{d}{d_{0}}+X_{\sigma }, \end{array}$$

where PL(*d*) is the path loss at distance *d* between transmitter and receiver, PL(*d*_0_) is the average path loss at a reference distance is (*d*_0_), *υ* is the path loss exponent, and *X*_*σ*_ is a zero mean Gaussian distributed random variable with standard deviation *σ*.

### 4.3. Simulation Results

In order to evaluate and validate the proposed AMH scheme, the proposed AMH is compared along with IF-HMIPv6 [28] and SFMMA [29] methods. It is worth mentioning that both the IF-HMIPv6 and SFMMA methods have been discussed earlier in related works section. The performance metrics which are considered to evaluate the proposed AMH scheme and state of the art (IF-HMIPv6 and SFMMA) are total average handover latency during simulation time with (HTTP, FTP, voice, and video) applications, handover signaling overhead cost, binding update signal cost, and the impact of both MN's speed and number on packet loss metric. In order to capture the selected metrics, five MNs have been selected randomly from each particular subnet in a constructed simulation scenario. It should be noted that each of the presented simulation results represents the average of 10 simulation runs in order to achieve a highly reliable performance evaluation procedure. Moreover, the confidence interval, that is, extremely valuable for any performance evaluation process, arrange of 95% was configured by utilized ANOVA single factor statistical method. Thus, this confidence interval range is used to estimate the true population value for ANOVA statistic.

#### 4.3.1. Total Handover Latency with HTTP, FTP, Voice, and Video Applications

In order to illustrate the impact of the proposed AMH scheme on the obtained handover latency during the MN roaming process, the handover latency has been captured with different applications and compared with other proposed methods. Four different kinds of applications are utilized which are categorized as normal network applications (HTTP and FTP) and as real time applications (voice and video). The main point of selecting these four different kinds of traffic is to show the effect of increasing the traffic rate on the handover process with each of the proposed AMH schemes and the state of the art. In other words, when the rate of packets/sec increases this will contribute negatively by increasing the MAC load, channel congestion, and the contention in the channel. Basically, changing traffic type increases the load on the link layer in order to satisfy the traffic requirements. This can be observed especially with real time applications (voice and video) which require a QoS which is normally tackled by MAC of each MN and AP. Therefore, the handover latency has been collected in each of the selected applications with the proposed AMH scheme, IF-HMIPv6, and SFMMA methods.

**Figure 10:**
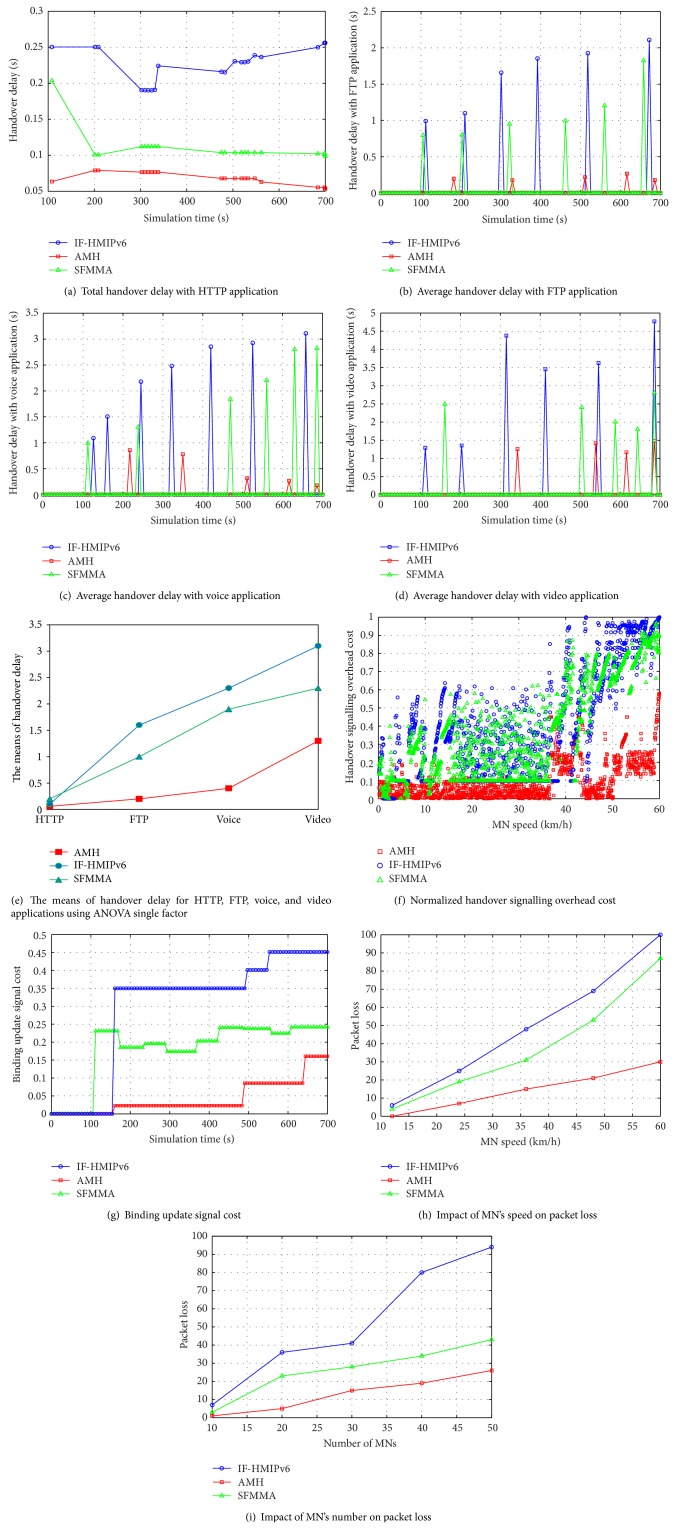
AMH evaluation metrics.

The total handover latency occurred during simulation time with browsing HTTP application in the proposed AMH scheme, IF-HMIPv6, and SFMMA as illustrated in comparison form in Figure 10(a). The handover latency capture began after 100 seconds and continued until the end of simulation time. The handovers were executed during this time. This is due to the fact that, during the first few seconds of running a simulation process, which is well known as the simulation warm-up process, not all simulation settings have been completed yet. Therefore, in the simulation settings, the capturing results process is configured to begin 100 seconds from the time simulation is executed.

It is clear from Figure 10(a) that the proposed AMH scheme is better able to decrease the total handover latency in comparison with IF-HMIPv6 and SFMMA methods. More precisely, the first time at second 100 when the handover was performed the latency in AMH, IF-HMIPv6, and SFMMA was (0.06, 0.25, and 0.2 seconds), respectively. It is obvious that the lowest handover latency was obtained using the proposed AMH scheme. Moreover, during the simulation time the handover latency in the AMH scheme ranged between 0.05 and 0.07 seconds which is considered low latency. On the other hand, the obtained latency range was between 0.19 up to 0.25 seconds and 0.09 up to 0.2 seconds in IF-HMIPv6 and SFMMA, respectively.

In contrast, Figure 10(b) depicts the average handover latency obtained each time the handover process occurred between MN and APs while FTP applications were run between MN and CN. In another words, the handover latency that occurred during a number of handovers performed by the MN during roaming process has been calculated as an average out of 10 simulation runs with FTP application. Figure 10(b) shows the average handover latency obtained by the AMH scheme during 5 handovers during simulation time with FTP application. The obtained latency with AMH scheme was 0.19, 0.17, 0.21, 0.26, and 0.17 Sec during simulation scenario time. The obtained average handover latency during the execution the FTP application with IF-HMIPv6 and SFMMA is 0.79, 0.8, 0.95, 0.99, 1.2, and 1.82 sec and 0.99, 1.1, 1.65, 1.85, 1.92, and 2.1 sec, respectively, based on 6 handovers. From the results in Figure 10(b) it can be seen that the handover latency with FTP application increased compared with the HTTP application. This is because the packet rate with FTP application increased compared to the HTTP which contributed to increasing the average handover latency. Furthermore, it can observed that the proposed AMH scheme could save the average obtained handover latency with FTP application, a maximum value of 0.26 sec compared with IF-HMIPv6 and SFMMA 1.82 and 2.1 sec, respectively.

Looking at Figure 10(c), the average handover latency obtained when the MN runs on the voice applications with CNs during its roaming process is illustrated. As can be seen, the collected average handover latency is depicted with (5, 6, 7) handover processes performed using AMH scheme, IF-HMIPv6, and SFMMA methods, respectively, with voice application. It is obvious that the handover latency with voice application increased sharply after 100 sec with both IF-HMIPv6 and SFMMA during the handover process. However, the AMH scheme could successfully keep the handover latency at an average value that satisfies the voice application requirements. More precisely, by looking at Figure 10(c) it can be seen that the first handover occurred after 100 seconds with both IF-HMIPv6 and SFMMA methods with delays of 0.99 and 1.09 sec, respectively, whereas the obtained delay with the AMH scheme was 0.85 sec after 217 seconds. The handover delay kept increasing with both IF-HMIPv6 and SFMMA methods during simulation time rising to 1.3, 1.84, 2.2, 2.8, and 2.82 and 1.50, 2.18, 2.48, 2.85, 2.92, and 3.10 sec, respectively.

On the other hand, the handover delay using the proposed AMH scheme continuously deceased to 0.77, 0.31, 0.26, and 0.17 sec as shown in Figure 10(c). It is worth mentioning that the proposed AMH scheme essentially contributes to decreasing the handover delay with voice application compared with IF-HMIPv6 and SFMMA methods. Hence, the QoS voice application requirements performed in the session by MN are satisfied during the handover process with the AMH scheme with low time delay. Accordingly, the proposed AMH scheme could optimally achieve its objectives, by maintaining a VoIP session with low handover delay compared to the state of the art.

The average handover latency required to execute video applications between MN and CNs has been captured during simulation time and is illustrated in Figure 10(d). Figure 10(d) shows the obtained handover latency compared to the latency of the AMH scheme, IF-HMIPv6, and SFMMA. It can be observed that the MN using the proposed AMH scheme tackled 4 times the handover process with delays of 1.25, 1.41, 1.16, and 1.47 sec at the simulation time 343, 539, 616, and 686 sec, respectively. On the other hand, the IF-HMIPv6 and SFMMA methods MN handled 5 and 6 times the handover process with delay times of 2.49, 2.4, 2, 1.8, and 2.8 and 1.29, 1.35, 4.38, 3.45, 3.62, and 4.76 sec at the simulation time 161, 504, 588, 644, and 686 and 112, 203, 315, 413, 546, and 686 sec, respectively.

As can be seen from the results in Figure 10(d), the handover delay during simulation time was obviously not constant especially with SFMMA method with high handover delay compared with the AMH scheme and IF-HMIPv6. This is due to the fact that the handover process with video traffic is considered a heavy real time application which consequently has a negative effect on the obtained handover delay. In other words, when the packet rate transferred per second increased each time the MN sends or receives video traffic with CN during the time a handover process is performed by the MN, the handover latency increased due to the MAC load which was mentioned earlier in this section. Moreover, the MN in SFMMA method relies on LMA to search for a possible candidate to direct this traffic flow in addition to the existing time delay of configuring the home network prefixes (HNP) between MN, MAG, and LMA which increases the total handover delay.

On the contrary, the obtained handover delay using IF-HMIPv6 method during video application was high compared with the proposed AMH scheme in spite of introducing a new way to formulate the new CoA in the MAP. This is because the CoA registration process is still visible in the MAP side during MN roaming in addition to the time consumed waiting to obtain the new CoA from the MN which is still valid as the IPv6 address of the MN changes when visiting different subnets. Due to the aforementioned reasons, the obtained handover delay with video application using both IF-HMIPv6 and SFMMA methods increased compared with the proposed AMH scheme.

In order to validate the presented handover delay results, ANOVA single factor method was utilized.  Figure 10(e) compares the average handover delay of AMH, SFMMA, and IF-HMIPv6 using ANOVA single factor for HTTP, FTP, voice, and video applications. The result indicates that AMH has the lowest means compared with the other two approaches. The illustrated validation results indicate that the means of AMH, SFMMA, and IF-HMIPv6 are 0.06, 0.1, and 0.2 sec, respectively, for HTTP application with *F* value of 372.47 and *p* less than 1% level of significance. In contrast, the means of handover delay for FTP application of AMH, SFMMA, and IF-HMIPv6 are 0.2, 1, and 1.6, respectively, with *F* value of 20.88 and *p* less than 1% level of significance. The obtained means of handover delay for voice application of AMH, SFMMA, and IF-HMIPv6 are 0.4, 1.9, and 2.3 sec, respectively, with *F* value of 11.66 and *p* less than 1% level of significance.

Finally, the means of handover delay for video application of AMH, SFMMA, and IF-HMIPv6 are 1.3, 2.3, and 3.1 sec, respectively, with *F* value of 4.03 and *p* less than 5% level of significance. These results suggest that the AMH scheme has lower means than the other two methods. The implication is that the method may be more efficient in reducing handover delay in HTTP, FTP, voice, and video applications than the other two methods as shown in Figure 10(e). Thus, the applied ANOVA single factor validation method significantly reflects the credibility of the proposed AMH scheme.

Consequently, it can be concluded from the presented handover latency results of HTTP, FTP, voice, and video applications that the AMH scheme could achieve the lowest handover delay with each particular application compared with the other proposed methods. This is no surprise since the proposed AMH scheme utilizes an advanced mobility model with a unique IPv6 address. There are two main advantages achieved by the AMH scheme. First, the ability to eliminate the time delay spent in obtaining a new CoA each time an MN visits a new network with a different subnet. To this must be added the time consumed in performing the handover in order to change the newly obtained AR's IPv6 address in each HA and CN routing entry. Thus, instead of the MN engaging during handover process by obtaining a new CoA and informing the HA and CN of the change in IPv6 address after the link layer handover which causes high handover latency, NAP tackles the registration process with a unique IPv6 address. Hence, the AMH scheme in general contributes essentially to decreasing the total handover delay.

#### 4.3.2. Handover Signalling Cost

In order to investigate the performance of AMH scheme in terms of reducing the cost of signaling overhead of handover procedure, in this section the results of total signaling cost and BU cost are presented and discussed.  Figure 10(f) illustrates the impact of MN's speed on the cost of handover signaling overhead of handovers which are performed employing AMH scheme, IF-HMIPv6, and SFMMA methods during simulation time. Normally, during handover process a location update procedure is performed which consists of several costs such as the binding update cost generated due to MNs mobility and the periodic binding refresh cost generated due to the expiration of the binding lifetime. By increasing the movement speed, the time required to process the aforementioned mobility handover process will be increased which subsequently increases the total signals overhead. For this reason, the impact of MN's speed on mobility handover signaling overhead is investigated in this section.

It can be observed from the results presented in Figure 10(f) the behaviour of signaling cost of performed handovers which was increasing similarly to MN's speed. Obviously, the proposed AMH scheme performed the best by saving the cost of signals which are associated with handover process in low average cost by the time the MN increases the movement speed compared with the state of the art. Regrettably, the average handover signaling overhead cost is the highest utilizing IF-HMIPv6 method which is increasing similar to the speed. In contrast, it can be observed that SFMMA method performed better than IF-HMIPv6 by obtaining lower signaling overhead cost.

The reason behind this is that the time the MN requires to perform more signals with other network components (MAP, MAG, and LMA) leads to establish a lifetime parameter for every particular signal; when times expires, the signal must be resent once again. In other words, with every handover signal performed there is a lifetime associated whereby decreasing this time as a way to avoid higher delay results in rising signal refreshment costs. Therefore, the proposed AMH scheme significantly decreased the number of signals which are triggered by the MN side to be combined into one developed AMH message. To this end, this dictated the tremendous improvement in decreasing the total handover signaling overhead cost using proposed AMH scheme.

On the other hand, the cost of binding update signal messages is collected employing the AMH scheme, IF-HMIPv6, and SFMMA methods as presented in Figure 10(g). Specificity, the cost of a BU signal message is calculated during simulation time at the time handovers which are performed by MN with APs in the simulated area. It is obvious that by the time handovers started after 100 sec of simulation time the BU cost started increasing slightly using the AMH scheme in contrast to the state of the art which sharply increased. As mentioned earlier in this section, when the MN is engaged with the BU signal message process it takes on the responsibility of the BU refreshing process which was performed due to the expiration of the binding lifetime. Hence, the obtained BU cost using IF-HMIPv6 is the highest followed by SFMMA method which utilizes media independent handover function (MIHF).

The implementation of MIHF of IEEE 802.21 standard in SFMMA method provides more improvement in the handover process compared with IF-HMIPv6 which applies the concept of HMIPv6 protocol. Although MIHF defines three different services, media independent event service (MIES), media independent command service (MICS), and media independent information service (MIIS), the MN is still associated with the network link changing process when the handover is triggered by the MN itself in SFMMA method. In contrast, the proposed AMH scheme omitted any interaction between the MN and any of the network components, except triggering the development of the AMH message which is embedded into the association request frame from the MN to selected APs.

#### 4.3.3. Packet Loss

The packet loss performance metric is investigated and evaluated in this section as a way of identifying the level of improvement that was achieved by decreasing the disconnect time due to the handover process employing the proposed AMH scheme. In order to achieve an accurate packet loss metric evaluation, the impact of MN's speed and its number are collected and presented in Figures 10(h) and 10(i), respectively.  Figure 10(h) presents the packet loss by the time the MN increases its speed (km/h). It is obvious from the graph of packet loss's behaviour that at the initial MN's speed, the packet loss obtained by operating the AMH scheme is equal to zero, and almost the same for both IF-HMIPv6 and SFMMA methods with low loss ratio. In this regard, it can be said that the MN with low speed has adequate time to establish the new connection with selected APs and to redirect the traffic flow of the MN to NAP. Therefore, the packet loss experienced a delay at 12 km/h which is the time required to send BU to the HA which is already eliminated in the proposed AMH scheme by elaborating the developed AMH message. Thus, the AMH scheme initially obtained zero packet loss compared to the state of the art.

As the speed increases, the packet loss ratio increased up to a maximum at 60 km/h speed using IF-HMIPv6 followed by SFMMA method compared to the proposed AMH scheme. AMH performed the best by efficiently decreasing the packet loss ratio. The important point to note here is that the time which is available to complete the processes of obtaining CoA, registration, and BU procedures was shorter as the MN's speed increases. Subsequently, a greater packet loss ratio was experienced as the MN speed was increasing. An additional weakness associated with increasing MN's speed is that the default nature of AP's channel accessibility decreases in WLANs based on IEEE 802.11b due to contention which occurs as speed increases. Accordingly, it can be possible that the MN does not obtain access over the wireless channel of the AP immediately after sending the association request frame; if not, it will be delayed. Regrettably, at higher speeds, wireless access contention increases, resulting in packet loss. In spite of the aforementioned high speed consequences, the AMH scheme could achieve lower packet loss ratio, compared to the state of the art, by keeping the value of packet loss at acceptable levels with low handover delay.

On the other hand, Figure 10(i) depicts the packet loss observed for a different number of MNs at a constant speed of 5 meters per second. When the handover is performed at a constant, low speed, the MN has sufficient time to execute CoA obtaining and registration processes. This implies that packet loss arises only during the time that the sending BU procedure to the HA is processed. Therefore, proportionally, when the number of MNs increases, packet loss increases due to the time delay experienced by the BU packet in order to reach its HA with a high number of MNs performing handovers.

In light of the above discussion, it can be observed that the AMH scheme can efficiently decrease the packet loss ratio as the number of MNs increases. In other words, with a maximum of 50 MNs, the AMH scheme obtained an average packet loss of 26 packets; the loss was 94 and 43 packets using IF-HMIPv6 and SFMMA methods, respectively. The reason is that the proposed scheme efficiently decreased the mobility handover delay by introducing developed AMH messages (AMH1: within the same subnet; AMH2: within different subnets with the same global routing prefix; and AMH3: within different subnets and different global routing prefixes). These AMH messages are devised to perform mobility handovers in advance of the handover in the link layer which suspends the high delay associated with the BU procedure. In addition, proposing unique IPv6 addresses to be utilized by MIPv6 protocols which are configured to all MNs functioning under the AMH scheme omits the procedures which belong to the CoA process. To this end, the AMH scheme efficiently decreased the packet loss ratio with respect to two impacts, MN's speed, and number compared to the state of the art.

## 5. Comparison of Main Handover Protocols Features

A critical comparison in terms of certain parameters is conducted between the well known mobility management handover protocols and presented by Table 10. It is clear that our proposed AMH scheme could achieve the best rating in terms of improving the main features in mobility-based management protocols. The main point to highlight here is that our proposed AMH scheme could efficiently decrease the mobility handover delay by elaborating an advanced handover in addition to the developed unique IPv6 address. Thus, the signalling cost was reduced; afterwards it was reflected as an improvement achieved with packet loss of running applications.

## 6. Conclusion

In this paper, we proposed an advanced mobility handover scheme to perform the network layer handover prior to its normal time (before the handover in link layer has been processed). These AMH scheme's processes were carried out by the next AP (NAP) after the MN triggers the developed AMH message that was embedded with association_req_ frame during the association process with NAP. Thus, the handover in the network layer level was performed by NAP on behalf of the MN while it was still communicating with the current AP. Moreover, a unique IPv6 address was developed in this paper as a way to keep the IPv6 address of the MNs during the simulated scenarios unchanged. In other words, during the time the MNs were visiting other wireless network providers with different subnets or different global routing prefixes, the MNs were not required to obtain a CoA from the visited networks. This was achieved by creating three main fields in IPv6 address format, which are global routing prefix, AP-ID, and MN-ID fields. Furthermore, three tables identified as neighbour AP and MN-ID_temp_ tables in APs, MN routing table in ARs, and Local MN table in HAs were cached. Employing these tables, routing and MN's IPv6 address registration processes became more dynamic in updating the ARs' IPv6 address during MN roaming. Therefore, numerical analysis has proven that by using the proposed AMH scheme, many of the costs and time delays that were normally associated to the handover process have been suppressed. Extensive and fair simulation results show that, compared to the other proposed methods IF-HMIPv6 and SFMMA, our AMH scheme performed better in terms of total and average handover latency with (HTTP, FTP, voice, and video) applications. Moreover, in terms of handover signaling overhead cost, binding update signal cost, and the impact of MN's speed in addition to the number on packet loss metric our AMH scheme performed the best. We are currently working on development of a prediction algorithm to reduce the handover latency in heterogeneous wireless networks.

## Figures and Tables

**Table 1:** Content of modified association request frame send by MN and AMH procedure process by next AP.

Body information	AMH
Default information + MN IPv6 address + (01)	Reserve memory space, establish MN-ID_temp_, and execute AMH1
Default information + MN IPv6 address + (10)	Reserve memory space, establish MN-ID_temp_, and execute AMH2
Default information + MN IPv6 address + (11)	Reserve memory space, establish MN-ID_temp_, and execute AMH3

**Table 2:** Data updating in each of AP and AR after AMH1 message.

AP2 entry table after T2	AR1 entry routing table after T2
2001:1:1:1:1::1/80 + MN-ID_temp_ (1)	2001:1:1:1:1::1/80 + 2001:1:1:1:2::/80

**Table 3:** Data updating in each of AP, AR, HA, and CN after AMH2 message.

AP3 entry table after T2	AR2 entry routing table after T2	HA&CN entry routing table after T2
2001:1:1:1:1::1/80 + MN-ID_temp_ (1)	2001:1:1:1:1::1/80 + 2001:1:2:1:1::/80	2001:1:1:1:1::1/80 + 2001:1:2:1::/64

**Table 4:** Data updating in each of AP and AR with different global routing prefix, HA, and CN after AMH3 message.

AP5 entry table after T2	AR3 entry routing table after T2	HA&CN entry routing table after T2
2001:1:1:1:1::1/80 + MN-ID_temp_ (1)	2001:1:1:1:1::1/80 + 2002:1:1:1:1::/80 + MN-ID	2001:1:1:1:1::1/80 + 2002:1:1:1::/64

**Table 5:** The traffic model for HTTP application.

Parameters	Value
Page interarrival times	Exponentially distributed with mean 60 seconds
Page properties	1000 bytes of text with 5 medium images
Image properties	Size randomly picked with a uniform distribution on [500, 2000] bytes

**Table 6:** The traffic model for FTP application.

Parameters	Value
File transfer	50% upload, 50% download
Interrequest file time	Exponentially distributed with mean 720 seconds
File size	5000 byte

**Table 7:** The traffic model for voice application.

Parameters	Value
Time spent in silence mode by called and calling party	Exponentially distributed with mean 0.65 seconds
Time spent in speech mode by called and calling party	Exponentially distributed with mean 0.352 seconds
Voice frame per packet	1
Bit generation rate	64 kbps
Type of service (ToS)	Best effort (high priority)
Compression voice packet delay	0.02 second

**Table 8:** The traffic model for video application (MPEG-2).

Frame interval time	25 frames/sec
Frame size (video resolution)	352 × 288 pixels
Bit generation rate	177.4 (low motion) to 709.6 kbps (high motion)
Bits per pixel	24
Type of service (ToS)	Best effort (high priority)

**Table 9:** Simulation parameters.

Parameters	Value
Simulation time	700 s
Simulation area	2300 × 1300 m
Mobility model	Linear and rectangle models
Number of MN	50 nodes
MN speed	1–18 m/s
Transmitted power	14 dbm
Transmission coverage range of each AP	300 m
Distance between two APs	500 m
Maximum packet generation rate	Maximum 800 packet/second
Maximum packet size	1000 byte
Channel bandwidth	11 Mbps
MAC protocol	IEEE 802.11b

**Table 10:** Comparison Between Main Mobility Management Protocols and Proposed AMH Scheme.

Parameters	MIPv6	HMIPv6	FMIPv6	PMIPv6	Proposed AMH
Complexity	Medium	High	High	Medium	Low
Handover Delay	High	High	Low	Medium	Very Low
Scalability	Medium	Medium	Medium	Low	High
Packet Loss	High	Medium	Medium	Medium	Low
Mobility	Host-Based	Host-Based	Host-Based	Network-Based	Host-Based
Signalling Overhead	High	Medium	High	Medium	Low
Operating Layer	Network Layer	Network Layer	Network Layer	Network Layer	Link and Network Layers
Mobility scope	Global	Local	Global/(local)	Local	Global/(local)
Handover Management	Yes (limited)	Yes	Yes	Yes	Yes
Location Management	Yes	Yes	No	Yes	No
Required Infrastructure	HA	HA, MAP	HA, optimised AR	LMA, MAG	HA, optimised AP, AR
Modification on MN	Yes	Yes	Yes	—	Yes
Route Optimization	Yes	Yes	—	—	Yes
Movement Detection	Required	Required	Required	Performed by L2	Performed by L2
